# Hepatitis B virus core protein with hot-spot mutations inhibit MxA gene transcription but has no effect on inhibition of virus replication by interferon α

**DOI:** 10.1186/1743-422X-7-278

**Published:** 2010-10-20

**Authors:** Yu Zhijian, Huang Zhen, Zhang Fan, Yang Jin, Deng Qiwen, Zeng Zhongming

**Affiliations:** 1 Department of Infectious Diseases, the Affiliated Shenzhen Nanshan Hospital of Guangdong Medical College, Shenzhen, China; 2 Department of of Infectious Diseases, the Affiliated DongHu Hospital of Guangdong Medical College, Shenzhen, China

## Abstract

It has been reported that hepatitis B virus (HBV) core protein (HBc) can inhibit the transcription of human interferon-induced MxA gene. In this study, we investigated whether HBc protein mutations at hot spots (L60V, S87G and I97L) could still inhibit MxA transcription and the potential significance of this inhibition in virus replication in vitro. Our data indicated that the IFN-induced MxA mRNA expression level and MxA promoter activity was significantly down-regulated by mutant protein of HBc(I97L), compared to WT and the other two mutated HBc proteins(L60V or S87G). However, in Huh7 cells stably expressing WT or the mutated HBc proteins (L60V, S87G or I97L), IFN-α could inhibit the extra- and intracellular HBV DNA level and HBsAg secretion to a similar level compared to that in cells transfected with control plasmids. In conclusion, HBc protein with I97L mutation may play an especial role in suppressing the transcription of MxA gene. Moreover, the inhibitory effect on MxA gene transcription by the WT or mutated HBc proteins (L60V, S87G and I97L) has no impact on inhibition of HBV replication by IFN-α in Huh7 cells. The clinical significance of the inhibitory effect of MxA gene transcription by HBc protein requires further study.

## Introduction

Many studies have indicated that hepatitis B virus (HBV) core gene mutations are significantly associated with hepatitis activity in patients with chronic hepatitis B (CHB) [1-4]. In addition, gene mutations in the precore/core region of HBV occur more frequently in patients with severe or fulminant hepatitis compared to asymptomatic carriers and those with acute self-limited hepatitis [1-3]. Several investigations have shown that the substitutions L60V, S87G and I97L in the HBV core antigen (HBcAg, referred to as the HBc protein) were the most frequent in patients with CHB, and HBV with these "hot-spot" mutations show different characteristics in replication cycle *in vitro *compared to the wild-type strain [4-9]. Moreover, *in vivo *infection with full-length HBV strains carrying these hot-spot mutations could alter the immune recognition sites of HBc protein thereby eliciting or evading immune clearance [4]. Recently, multiple reports have demonstrated that HBc protein can have various effects on expression and transcription of some intracellular cytokines and proteins [10-12]. However, it is unknown whether HBc protein with hot-spot mutations would play a different role compared to the wild-type (WT).

Intracellular transcription and expression of human MxA protein is specifically dependent on induction by type I interferon (IFN), and furthermore, MxA protein plays an important antiviral role as a downstream mediator of type I interferon [13-15]. Human MxA protein, a GTPase, can inhibit the replication of a wide range of negative- and positive-strand RNA viruses as well as HBV [14,16,17]. Recently, HBc protein has been shown to trans-suppress IFN-induced gene expression and to down-regulate the promoter activity of the MxA gene by direct interaction with the IFN-stimulated response element (ISRE) sequence of the MxA promoter [12,18]. However, it is still unknown whether HBc protein carrying the hot-spot mutations has a different effect on transcription and expression of the MxA gene compared to the wild-type (WT). Furthermore, it remains to be elucidated whether the inhibition of MxA gene transcription by HBc protein influences the inhibition of HBV replication by IFN-α.

## Materials and methods

### 1. Plasmid constructs

The parental plasmid for pU19-1.24HBV was kindly provided by Dr. Mizokami [14]. The WT HBc gene was amplified from pU19-1.24HBV using the sense primer: 5'-GGGGCCTAAAA CTCAGACAACTATTG-3' and antisense primer: 5'-GCAAGCTATTGTGTGTTGG-3'. pCMV-HBc (WT), the expression vector for WT HBc protein, was constructed with the WT HBc gene inserted into pCMV-Tag1 (containing the Flag-tag, purchased from Stratagene Company) by standard procedures. Using pCMV-HBc (WT), the other three plasmids [pCMV-HBc (L60V), pCMV-HBc (S87G) and pCMV-HBc (I97L)] expressing HBc proteins with the substitutions L60V, I97L and S87G, respectively, were constructed using the Quick Change Site-Directed Mutagenesis Kit (Stratagene, USA) and the primers described previously [4]. pCMV-HBc (WT), pCMV-HBc (L60V), pCMV-HBc (S87G) and pCMV-HBc (I97L) were confirmed by sequencing and these vectors were able to express the HBc protein/Flag-tag fused protein. The pMxA550-Luc plasmid was constructed by insertion of the 550 bp minimal MxA gene promoter (+553, -10) in front of the luciferase gene of the pGL3 basic vector (Promega) as described previously [19]. *Renilla *luciferase vector was purchased from Promega.

### 2. Cell culture, transfection, harvest and measurement of Luciferase activity

First, the influence of the HBc proteins (WT and mutated) on the expression level of MxA mRNA in Huh7 cells was determined. Huh7 cells (2 × 10^5 ^per well) were seeded in 12-well plates, with 0.5 mL DMEM media containing 10% FBS per well (Gibco). After 24 h, Huh7 cells were transfected with 0.5 μg pCMV-HBc (WT), pCMV-HBc (L60V), pCMV-HBc (S87G), pCMV-HBc (I97L) and control DNA (pCMV-Tag1 and salmon DNA), respectively, using FuGene HD transfection reagent (Roche). After transfection for 48 h, culture medium was removed and fresh media with IFN-α (1000 IU/mL) was added and incubated for a further 8 h at 37°C. Cells were then collected for detection of HBc protein expression by western blot as described below, and used for extraction of total RNA by Trizol (Invitrogen). Levels of MxA mRNA were assessed by real-time PCR as described below. In addition, the influence of the HBc proteins (WT and mutated) on the promoter activity of the MxA gene was examined. Huh7 cells were seeded in 12-well plates at 2 × 10^5 ^cells per well and, after 24 h, pMxA550-Luc (0.25 μg) was co-transfected in Huh7 cells with 0.5 μg pCMV-HBc (WT), pCMV-HBc (L60V), pCMV-HBc (S87G), pCMV-HBc (I97L) and control DNA (pCMV-Tag1 and salmon DNA), respectively, using FuGene HD (Roche). After 48 h, the culture medium was removed and fresh media with IFN-α (1000 IU/mL) was added and incubated for a further 8 h at 37°C. The cells were then lysed and collected and the luciferase activity in the cellular lysate was measured by Glomax (Promega) according to the manual of the Dual-Luciferase Reporter Assay System (Promega). *Renilla *luciferase vector (40 ng) was used to control the transfection efficiency.

Moreover, we wished to examine whether the inhibition of MxA transcription by HBc protein would decrease the inhibitory effect of IFN-α on HBV replication. pCMV-HBc (WT), pCMV-HBc (L60V), pCMV-HBc (S87G), pCMV-HBc (I97L) and pCMV-Tag1 were transfected into Huh7 cells as described above, using 0.5 μg of DNA. Subsequently, transfected cells were selected with neomycin (100 mg/mL). After 2 weeks, the positive cells transfected with the above vectors were identified to express WT or the mutated HBc protein by western blot as described below. The cells stably expressing WT or mutated HBc protein, and control cells transfected with the empty vector (pCMV-Tag1), were seeded in 10 cm dishes respectively. After 24 h, pU19-1.24HBV (10 μg) and *Renilla *luciferase vector (60 ng) were co-transfected into the Huh7 cells. After transfection for 24 h, culture medium was removed and fresh media with IFN-α (1000 IU/mL) was added and incubation continued for a further 24 h at 37°C. Then, the relative MxA and GAPDH mRNA levels, HBV DNA and hepatitis B surface antigen (HBsAg) were assessed as described below.

### 3. Western blot analysis

For SDS-PAGE, cells were lysed in lysis buffer (Cell Signaling Technologies), and the protein content measured using the Bradford technique (Bio-Rad). Twenty-one micrograms of total protein was boiled in SDS loading buffer and loaded onto the gel. Proteins were then transferred to a nitrocellulose membrane (Amersham). After incubation with blocking solution (Invitrogen), the blots were incubated with the antibody (anti-Flag, 1:3000; poly-anti-rabbit β-actin, 1:500). The monoclonal anti-Flag antibody was purchased from the Invitrogen Company and poly-anti-rabbit β-actin antibody was purchased from the Santa Cruz Biotechnology. Detection was carried out using a horseradish peroxidase-conjugated secondary antibody (1:2000) (Santa Cruz Biotechnology) and chemi-luminescence development was carried out (Pierce).

### 4. Real-time analysis of MxA mRNA and HBV DNA levels

For real-time analysis of MxA mRNA detection, 500 ng of total RNA were converted to cDNA using TaqMan reverse transcription reagents (Applied Biosystems, Foster City, CA, USA). Real-time PCR to detect MxA mRNA and GAPDH mRNA levels was performed with the reported methods, DNA primers and probes[20]. Relative amount of MxA mRNA level were reported as number of folds of MxA mRNA copies relative to the expression of GAPDH. Increasing folds= mean relative amount (experimental group)/mean relative amount (control group).

The extra- and intracellular HBV DNA levels in the core particles were detected by real-time PCR analysis as previously described [16]. The culture supernatant and the cytoplasmic lysate of the transfected cells were digested with DNase I to remove plasmid DNA. Subsequently, HBV DNA from the core particles was extracted from 100 μL of supernatant or cytoplasmic lysate using a High Pure viral nucleic acid extraction kit (Qiagen) and quantified using the LightCycler-based real-time fluorescence quantitative PCR system [18,19]. The amount of extra- and intracellular core particles extracted from each plate of transfected cells was normalized using the expression of *Renilla *luciferase in the cytoplasmic lysate as an internal control. The decreased log_10 _value of HBV DNA level = [mean log_10 _value (control group) - mean log_10 _value (experimental group)].

### 5. Determination of hepatitis B surface antigen (HBsAg)

Extracellular HBsAg in the culture supernatant was detected with a commercial assay kit (Abbott Diagnostics). The percentage change = [mean (control group) - mean (experiment group)]/mean (control group).

### 6. Statistics

Results are reported as means ± standard deviation (SD). Differences among groups were tested for significance by one-way analysis of variance (ANOVA). Values of *P *< 0.05 were considered significant. All statistical calculations were performed with SPSS10.5 program.

## Results

### 1. Influence of HBc protein with hot-spot mutations on the MxA mRNA level and gene promoter activity in Huh7 cells

**Figure 1:**
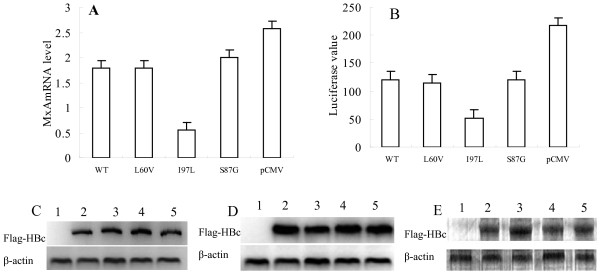
**The different effect of HBc proteins on the MxA mRNA level in Huh7 cells was assessed by real time-PCR (a) and the level of Flag-tagged HBc protein was analyzed by western blot using an anti-Flag antibody (c)**. The different effect of HBc proteins on MxA promoter activity was also examined by luciferase assay (b) and the level of Flag-tagged HBc protein was analyzed by Western blot using an anti-Flag antibody (d). In the stably-transfected Huh7 cells, the expression of the WT and mutated (L60V, S87G and I97L) HBc proteins were detected by western blot using an anti-Flag antibody (e). In (c), (d), and (e), lanes 1-5 represent the expression of Flag-HBc proteins and β-actin in Huh7 cells transfected with pCMV-Tag1, pCMV-HBc (WT), pCMV-HBc (L60V), pCMV-HBc (S87G) and pCMV-HBc (I97L), respectively.

First, we compared the different effect of the HBc proteins (WT and mutated) on the MxA mRNA level in Huh7 cells respectively. Compared to control cells transfected with pCMV-Tag1, MxA mRNA expression induced by IFN-α in Huh7 cells transfected with WT and mutated HBc proteins (L60V, I97L and S87G) was decreased to 69.1%, 69.1%, 21.9% and 73.1%, respectively (Figure 1A), suggesting the L60V and S87G mutated proteins have a similar effect to WT (*p *> 0.05). In contrast, I97L HBc protein remarkedly decreased the level of MxA mRNA compared to WT (*p *< 0.01). Subsequently, we investigated the different effects on the promoter activity of MxA gene by the HBc proteins (WT and mutated). As described in Figure 1B, MxA gene promoter activity in Huh7 cells was influenced to different degrees by the HBc proteins (WT and mutated). Our data showed that the promoter activity of MxA gene in the Huh7 cells transfected with WT and mutated HBc proteins (L60V, I97L and S87G) was decreased to 55.2%, 53.3%, 24.1% and 55.9%, respectively (Figure 1B) compared to the control cells transfected with pCMV-Tag1. This result showed that I97L HBc protein could remarkably decrease the MxA promoter activity induced by IFN-α compared to the WT or other two mutated HBc proteins (L60V or S87G) (*p *< 0.01). It was consistent with the result on mRNA expression. The expression level of Flag-tagged HBc protein was analyzed by western blot using an anti-Flag antibody and the results showed no apparent differences in expression of the tagged fusion proteins (Figure 1C,D and 1E).

### 2. HBc and its three hot-spot mutants do not affect the inhibition of HBV replication by IFN-α

**Figure 2:**
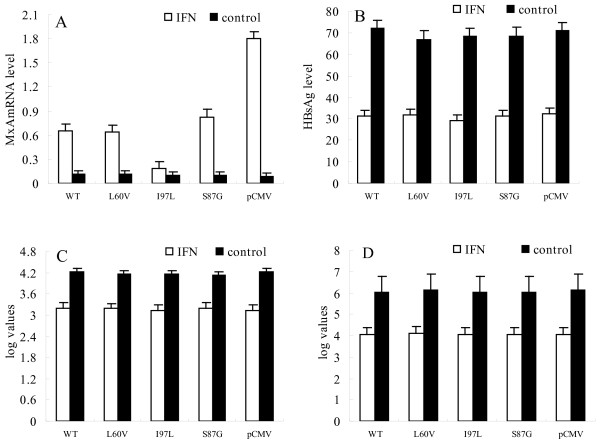
**pU19-1.24HBV was co-transfected with *Renilla *luciferase vector into Huh7 cells stably expressing wild-type HBc protein, L60V, S87G or I97L mutated proteins, as well as into control cells stably transfected with empty vector**. MxA mRNA levels in these groups of cells were then detected by real time-PCR (a); the extracellular HBsAg level was assessed by abbot analysis (b) and the extra- and intracellular HBV DNA level (c and d respectively) was measured by real-time PCR.

In order to evaluate whether the inhibition of MxA transcription by HBc protein could decrease the inhibitory effect on HBV replication induced by IFN-α, pU19-1.24HBV was co-transfected with *Renilla *luciferase vector into Huh7 cells stably expressing WT HBc protein, L60V, S87G and I97L mutated proteins, respectively. The level of Flag-tagged HBc protein was analyzed by western blot using an anti-Flag antibody (Figure 1E). The MxA mRNA level was detected by real-time PCR. In the stable expression cells after addition of IFN-α, the MxA mRNA levels in the WT, L60V, I97L, S87G and pCMV vector only transfected cells were increased by 5.91-fold, 5.33-fold, 1.80-fold, 8.30-fold, and 20-fold respectively compared to those without IFN-α (Figure 2A). The extracellular HBsAg in these groups of cells was decreased to 58.2%, 54.5%, 57.4%, 50.6% and 54.4%, respectively (*p *> 0.05) (Figure 2B) compared to groups non-treated by IFN-α (Figure 2B); the decreased values of extracellular HBV DNA level were 2.00, 2.01, 1.99, 1.93 and 2.06 log_10_, respectively (*p *> 0.05) (Figure 2C); and the decreased values of the intracellular HBV DNA were 1.03, 1.03, 1.01, 0.94 and 1.09 log_10_, respectively (*p *> 0.05) (Figure 2D). We observed the similar result that WT and hot-spot mutant HBcs inhibitted MxA mRNA expression in stably transfected cells and mutant I97L has most dramatic effect (Fig 2A), this is consistent with our finding in transiently transfected cells (Fig 1). Our data also demonstrated that there is no significant change of HBsAg level, extracellular or intracellular HBV DNA after IFN-α induction among cells stably expressing WT, hot-spot mutant HBc or expressing no HBc, suggesting that neither WT HBc nor its three hot-spot mutants interfere the inhibitory effect on HBV replication caused by IFN-α.

## Discussion

IFN-α has been widely used in the treatment of CHB. The antiviral action of IFN-α is mediated by the induction and activation of at least three IFN-inducible proteins: 2,5-oligoadenylate synthetase (OAS), p68 protein kinase (PK) and the MxA protein [21]. Several *in vivo *studies have shown a lack of activation of the IFN system in patients with acute or chronic hepatitis B [10,11,21]. Recent *in vitro *studies have suggested that viral factors of HBV might play a direct role in the resistance to the antiviral action of endogenous or exogenous IFN, and multiple reports have demonstrated direct inhibition of the IFN-induced MxA protein expression by the HBc protein, through interaction with the MxA gene promoter [9,11,12]. Different replicative characteristics and clinical significance of full-length HBV strains carrying core protein hot-spot mutations (L60V, S87G and I97L) has also been reported [4-9,22]. However, it is still unknown whether HBc protein with these hot-spot mutations also has the inhibitory effects on IFN-induced MxA gene expression that are observed with WT. In our study, we showed that L60V and S87G HBc protein decreased the MxA mRNA level induced by IFN-α in Huh7 cells to a similar degree as WT. Moreover, compared with WT and the other two mutants (L60V, S87G), I97L HBc protein remarkably decreased the MxA mRNA level. Consistently, our data also showed that WT, L60V, S87G and I97L HBc protein downregulated the promoter activity of the MxA gene induced by IFN-α. In particular, I97L HBc protein remarkably inhibited the MxA promoter activity compared to L60V, S87G or WT. Our result demonstrated in vitro that the HBc hot-spot mutants also inhibited the mRNA level induced by IFN-α and I97L showed significantly more inhibitory effect than that of WT. These results indicated that HBc protein with certain hot-spot mutations might have a different influence on MxA transcription to that of WT. From this study, we suppose that the mutated HBc proteins might down-regulate MxA gene transcription by inhibiting promoter activity induced by IFN-α in a similar way to that reported for WT HBc protein[18], but the special inhibitory role of I97L HBc on MxA transcription in Huh7 cells need to be further studied.

Many evidences indicated that the IFN-inducible MxA protein played a key role in the antiviral action of IFN-α against various RNA viruses [13-15]. Recently, Gordien *et al *reported that MxA was able to inhibit HBV replication by inhibiting the nucleocytoplasmic export of viral mRNAs [16,17]. Another study in HBV and HBV/MxA transgenic mice showed that MxA expression was sufficient to moderately downregulate the expression of viral proteins and reduce the synthesis of HBV DNA, without affecting the steady-state levels of HBV RNAs[23]. However, although several studies (including the present study) have demonstrated the inhibitory effect on transcription and expression of the MxA gene by HBc protein, it is still necessary to clarify whether this inhibitory effect of HBc protein on MxA gene transcription can influence the antiviral action of IFN-α on HBV replication. Our data showed that, in Huh7 cells stably expressing WT HBc protein or mutated proteins (L60V, S87G and I97L), IFN-α inhibited the extra- and intracellular levels of HBV DNA, and HBsAg secretion to a similar degree to that in the cells transfected with control plasmids. This suggests that the inhibitory effect on MxA gene transcription by WT or mutated HBc protein may have no impact on inhibition of HBV replication by IFN-α. In fact, Rang et al demonstrated that IFN-α could also suppress HBV replication in MxA-deficient HEp2 cells, indicating that MxA might be not essential for this activity [24]. Frese et al also showed that IFN-α inhibited hepatitis C virus subgenomic RNA replication via an MxA-independent pathway [25]. Therefore, we consider that IFN-α may inhibit HBV replication independently of the MxA pathway in Huh7 cells.

## Conclusion

In conclusion, our data show that HBc protein with hot-spot mutations (L60V, S87G and I97L) can also inhibit MxA gene expression and different mutations have different effect in Huh7 cells; compared to WT HBc protein and the other two mutated proteins (L60V or S87G), HBc protein with the I97L mutation plays an especial role in suppressing transcription and promoter activity of the MxA gene induced by IFN-α. Moreover, our results reveal that the inhibition of MxA gene transcription by WT HBc or its hot-spot mutants has no impact on inhibition of HBV replication by IFN-α, indicating MxA may be independent of inhibition of HBV DNA replication by IFN-α. The clinical significance of the inhibitory effect on MxA gene transcription by HBc protein requires further study.

## Competing interests

The authors declare that they have no competing interests.

## Authors' contributions

DQ and ZZ conceived the study and DQ wrote the paper. YZ, HZ, ZF, YJ participated in the laboratory studies. All authors read and approved the final manuscript.
